# Supplementary material for: TGF-β Regulates Collagen Type I Expression in Myoblasts and Myotubes via Transient Ctgf and Fgf-2 Expression
Source: Cells. 2020 Feb 6;9(2):375. doi: 10.3390/cells9020375 (PMC7072622; doi:10.3390/cells9020375)
Supplement: Supplementary file 1 [file cells-09-00375-s001.pdf]

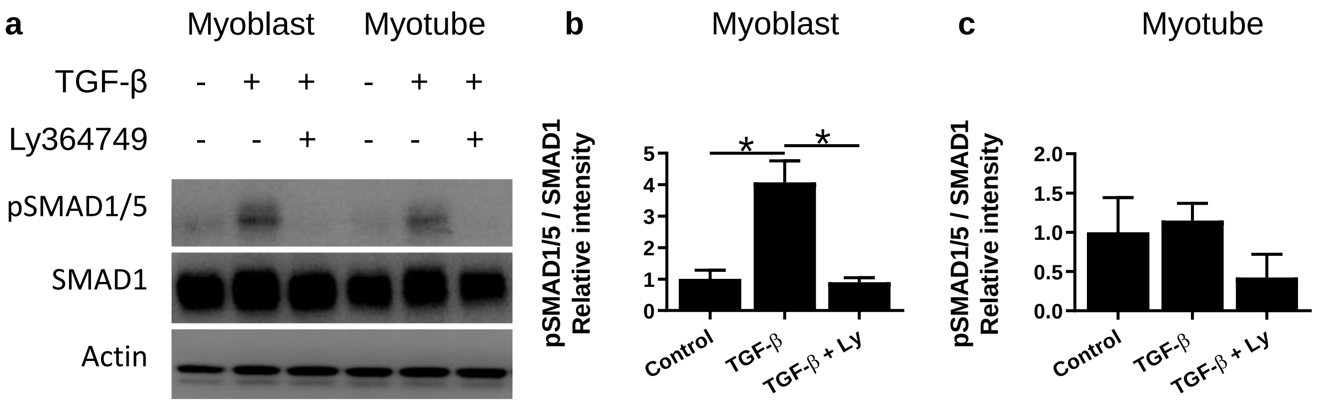

Figure S1. TGF-β supplementation results in SMAD1/5 phosphorylation in C2C12 myoblasts and myotubes. **a, b** Western blot quantification of SMAD1/5 phosphorylation in myoblasts, **c** SMAD1/5 phosphorylation in myotubes. SMAD1/5 phosphorylation was inhibited by TGF-β type I receptor chemical blocker Ly364749. Pan actin served as loading control. Phosphorylation levels are displayed as relative intensity of p-SMAD/total SMAD. Data were normalized to values of control condition. Error bars indicate standard error of the mean; * indicates significant difference at $P<0.05$; n=4 experiments per condition.
